# Disruption of c-MYC Binding and Chromosomal Looping Involving Genetic Variants Associated With Ankylosing Spondylitis Upstream of the *RUNX3* Promoter

**DOI:** 10.3389/fgene.2021.741867

**Published:** 2022-01-07

**Authors:** Carla J. Cohen, Connor Davidson, Carlo Selmi, Paul Bowness, Julian C. Knight, B. Paul Wordsworth, Matteo Vecellio

**Affiliations:** ^1^ Nuffield Department of Orthopaedics, Rheumatology and Musculoskeletal Sciences, University of Oxford, Oxford, United Kingdom; ^2^ National Institute for Health Research Oxford Comprehensive Biomedical Research Centre, Botnar Research Centre, Nuffield Orthopaedic Centre, Oxford, United Kingdom; ^3^ Wellcome Centre for Human Genetics, University of Oxford, Oxford, United Kingdom; ^4^ Division of Rheumatology and Clinical Immunology, Humanitas Clinical and Research Center - IRCCS, Rozzano, Italy

**Keywords:** ankylosing spondylitis, single nucleotide polymorphism (SNP), chromosome conformation capture (3C), RUNX3, c-Myc

## Abstract

**Background:** Ankylosing Spondylitis (AS) is a common form of inflammatory spinal arthritis with a complex aetiology and high heritability, involving more than 100 genetic associations. These include several AS-associated single nucleotide polymorphisms (SNPs) upstream of *RUNX3,* which encodes the multifunctional RUNT-related transcription factor (TF) 3. The lead associated SNP *rs6600247* (*p* = 2.6 × 10^−15^) lies ∼13kb upstream of the *RUNX3* promoter adjacent to a c-MYC TF binding-site. The effect of *rs6600247* genotype on DNA binding and chromosome looping were investigated by electrophoretic mobility gel shift assays (EMSA), Western blotting-EMSA (WEMSA) and Chromosome Conformation Capture (3C).

**Results:** Interrogation of ENCODE published data showed open chromatin in the region overlapping *rs6600247* in primary human CD14^+^ monocytes, in contrast to the Jurkat T cell line or primary human T-cells. The *rs6600247* AS-risk allele is predicted to specifically disrupt a c-MYC binding-site. Using a 50bp DNA probe spanning *rs6600247* we consistently observed reduced binding to the AS-risk “C” allele of both purified c-MYC protein and nuclear extracts (NE) from monocyte-like U937 cells. WEMSA on U937 NE and purified c-MYC protein confirmed these differences (*n* = 3; *p* < 0.05). 3C experiments demonstrated negligible interaction between the region encompassing *rs6600247* and the RUNX3 promoter. A stronger interaction frequency was demonstrated between the *RUNX3* promoter and the previously characterised AS-associated SNP *rs4648889*.

**Conclusion:** The lead SNP *rs6600247,* located in an enhancer-like region upstream of the *RUNX3* promoter, modulates c-MYC binding. However, the region encompassing *rs6600247* has rather limited physical interaction with the promoter of *RUNX3*. In contrast a clear chromatin looping event between the region encompassing *rs4648889* and the *RUNX3* promoter was observed. These data provide further evidence for complexity in the regulatory elements upstream of the *RUNX3* promoter and the involvement of *RUNX3* transcriptional regulation in AS.

## Introduction

### Background

Ankylosing Spondylitis (AS) is a form of inflammatory spondyloarthritis predominantly affecting the axial skeleton, which is characterised pathologically by enthesitis (Bridgewood et al., 2020). Extra-skeletal manifestations are also common in AS; these include inflammation of the gut (ranging from low-grade sub-clinical inflammation of the terminal ileum to overt inflammatory bowel disease - IBD), skin (psoriasis), and uveal tract (Stolwijk et al., 2015; Rizzo et al., 2017). AS was one of the first complex diseases in which a specific genetic effect was identified when its strong association with the major histocompatibility complex (MHC) immune response gene HLA-B27 was described nearly 50 years ago (Brewerton et al., 1973) (Schlosstein et al., 1973). However, it is clearly polygenic (Brown et al., 1997); even the MHC association is attributable to several alleles at more than one locus (Cortes et al., 2015) and more than 100 non-MHC genetic associations have now been suggested by genome-wide association studies (Burton et al., 2007; Reveille et al., 2010; Evans et al., 2011; Cortes et al., 2013). Shared genetic susceptibility factors undoubtedly contribute to the excess occurrence of psoriasis, IBD and uveitis not only in individuals with AS but also their relatives (Ellinghaus et al., 2016) (Robinson et al., 2016). One of the strongest non-HLA associations with AS is with the *RUNX3* (Runt-related transcription factor (TF) 3) locus. RUNX3 is involved in T-cell function and plays a key role in the development of CD8^+^ T-cells (Egawa et al., 2007). It also influences many other cells, including helper T-cells, innate lymphoid, tissue resident, mucosa and gut cells (Ebihara et al., 2015; Behr et al., 2018). We have recently demonstrated that AS-associated non-coding single nucleotide polymorphisms (SNPs) in an enhancer-like region upstream of *RUNX3* affect the binding of different factors: in particular the repressive nucleosome remodelling and deacetylase (NuRD) complex binds preferentially to the risk allele, while conversely interferon regulatory factor (IRF) five to the protective allele (Vecellio et al., 2021). However, the functional effects of these changes on gene transcription are still to be precisely determined. Our earlier observations were made in T-cells, but here we describe some of the functional effects of the lead AS-associated SNP in the vicinity of *RUNX3* (*rs6600247*, *p* = 2.6 × 10^−15^ (Cortes et al., 2013) that are more obvious in CD14^+^ monocyte-like cells than CD8^+^ T-cells. First, we evaluate the chromatin landscape surrounding *rs6600247* using the ENCODE database (https://genome.ucsc.edu/ENCODE/). Second, we demonstrate differential allelic binding of *rs6600247* to the c-MYC TF. Finally, we investigate the chromosomal architecture and physical interactions between AS-associated sequences in the enhancer-like region upstream of *RUNX3* and its promoter, showing a probable role of chromosome looping in the regulation of *RUNX3*.

## Methods

### Genotyping

DNA was extracted using the Qiagen AllPrep DNA/RNA Mini Kit (Qiagen Ltd., Manchester, United Kingdom) and genotyped for *rs6600247* using TaqMan SNP assay (custom order by Life Technologies, Paisley, United Kingdom), for the cells (obtained by the buffy coat) used in the functional studies.

### *In Silico* Investigation

We used the UCSC genome browser build hg19 and the Roadmap database [https://genome.ucsc.edu/ENCODE/] to investigate the epigenetic landscape of *rs6600247* upstream of the *RUNX3* promoter, which is strongly associated with AS (*p* = 4.2 × 10^–15^) (Cortes et al., 2013). Histone modifications and GeneHancer (a database of human regulatory elements and their inferred target genes) tracks were selected to evaluate regulatory elements and chromosome looping between promoters and enhancer regions (Fishilevich et al., 2017).

### Cell Lines, Culture and Primary Human Cell Isolation

Blood samples were obtained from AS patients with ethical approval (COREC **06/Q1606/139)** and informed patient consent. CD8^+^ T-cells and CD14^+^ monocytes were isolated from AS patients’ peripheral blood mononuclear cells (PBMCs) using a CD8^+^ T-cell or a CD14^+^ monocyte isolation kit (Miltenyi, Bisley, Surrey, United Kingdom), respectively. Jurkat, U937, CD8^+^ and CD14^+^ cells were resuspended at 1×10^6^/ml in pre-warmed Roswell Park Memorial Institute medium supplemented with 10% fetal bovine serum, penicillin/streptomycin and l-glutamine, and rested overnight. Cells were then harvested for experiments.

### Electrophoretic Mobility Gel Shift Assay

The impact of *rs6600247*, which lies in a c-MYC binding-site (Figure 2A), was assessed by EMSA. We designed DNA probes including either the protective T or the AS-risk variant C to evaluate the disruption of a c-MYC consensus motif. The DNA probes used in EMSAs (50-bp single-stranded biotinylated DNA probe incorporating *rs6600247*) were mixed and annealed at room temperature for 1 h. Probes were then incubated for 20 min with nuclear extracts (NE) obtained either from primary CD8^+^ T-cells or a monocyte cell line from histiocytic lymphoma (U937) stimulated with phorbol-12-myristate-13-acetate (PMA). The sequences of the synthetic single-stranded oligonucleotides are listed below:

C* s (sense): 5′-CTC​CAT​GAC​GCA​ATT​TGG​GCT​CCGTT​ATG​AGT​CAG​CTC​AAG​TAA-3′; T* s: 5′-CTC​CAT​GAC​GCA​ATT​TGG​GCT​CTGTT​ATG​AGT​CAG​CTC​AAG​TAA-3′; C* as (antisense): 5′-TTA​CTT​GAG​CTG​ACT​CAT​AACGGAG​CCC​AAA​TTG​CGT​CAT​GGA​G-3′; T* as: 5′-TTA​CTT​GAG​CTG​ACT​CAT​AACAGAG​CCC​AAA​TTG​CGT​CAT​GGA​G-3′.

(Underlined base highlights the position of *rs6600247*).

### Western Blotting - Electrophoretic Mobility Gel Shift Assay

DNA probes as for EMSA (above) were incubated with nuclear extract obtained from U937, CD8^+^ T-cells or purified c-MYC human recombinant protein (Abcam, ab169901 Cambridge, United Kingdom) as previously described (Allen et al., 2017) and separated on DNA retardation gels at 100 V on ice. The samples were transferred on to nitrocellulose membranes for Western blotting (WB), then blocked with 5% milk in Tris Buffer Saline +0.1% Tween (TBST) for 1 h at room temperature (RT) before incubating overnight at 4°C with the primary antibody for c-MYC (Santa Cruz Biotechnology sc-40, Dallas, Texas United States). Secondary goat anti-rabbit antibody (1:10,000 dilution) was added (1 h RT) and the membranes washed before Horse Radish peroxidase substrate (Thermo Fisher Scientific, Waltham, Massachusetts, United States) added for imaging. ImageJ (NIH) was used for quantifying WEMSA bands (Schneider et al., 2012).

### Chromosome Conformation Capture

Chromosome conformation capture (3C) was performed as previously described (Miele et al., 2006). Briefly, libraries were prepared as follows: 1.5 × 10^7^ of U937 or Jurkat cells were cross-linked with formaldehyde at 1% of the final volume. *Glycine* [0.125M] was used to quench cross-linking and cells were lysed in cold lysis buffer on ice using a Dounce homogenizer (Sigma Aldrich, Gillingham, United Kingdom). Cells were resuspended in specific restriction enzyme buffer (10 μL were kept as undigested control). The remaining samples were digested overnight at 37°C with 500 units of Sac1 (New England Biolabs, Hitchin, United Kingdom). Digestion was stopped by the addition of 10% sodium dodecyl sulfate incubated at 65°C for 30 min. T4 ligase (Ambion, Thermo Fisher Scientific, Waltham, Massachusetts, United States) was used to perform ligation for 4 h at 16°C. Proteinase K was added prior to reversal of cross-linking at 65°C overnight. Proteinase K was added to the undigested and digested controls saved earlier. DNA was purified using phenol-chloroform extraction, followed by ethanol precipitation. 3C template was resuspended in 500 ul H_2_O, while undigested and digested controls in 50 ul. The quality of the chromatin samples was assessed on agarose gels. Bacterial Artificial Chromosome (BAC) preparations were performed similarly as genomic controls. 3C PCR primers were designed along the same strand and same orientation to accomplish specific amplification across 3C ligation junctions. Full list of primers is available in Supplementary Table S1 and their genomic position relative to *RUNX3* is shown in Figure 3

We interrogated a genomic region upstream the *RUNX3* distal promoter, including few AS-associated SNPs in U937 (monocyte-like) and Jurkat (T-lymphocyte-like) cell lines. The bait was placed at the distal promoter (P2) with amplification primers at the AS-associated SNPs *rs6600247* and *rs4648889* along with three intergenic regions.

### Quantitative Real-Time Polymerase Chain Reaction

Total RNA from CD8^+^ and CD14^+^ cells was isolated with TRIzol (Invitrogen, Paisley, United Kingdom) and reverse transcribed with Superscript III (Invitrogen, Thermo Fisher Scientific, 168 Third Avenue, Waltham, Massachusetts, United States) to synthesise cDNA as previously described (Vecellio et al., 2018). The specific primers were: *RUNX3* sense (s): 5′-ACTCAG CAC CAC AAG CCA CT-3′; *RUNX3* antisense (as): 5′-GTC GGA GAA TGG GTT CAG TT-3′. Quantitative PCR was performed in triplicate and the 2−ΔCt method was used to calculate the expression of *RUNX3* relative to β-actin (ID Assay qHsaCED0036269, Bio-Rad Laboratories, Kidlington, United Kingdom).

### Historical Controls and RUNX3 Expression

*RUNX3* transcription in AS cases and controls was evaluated from previously published data derived from RNA-seq in PBMCs from 72 AS cases and 62 healthy controls and stratified for *rs6600247* (Li et al., 2017).

## Results

### Genomic Landscape Interrogation Suggests a Regulatory Role for *rs6600247*

**FIGURE 1:**
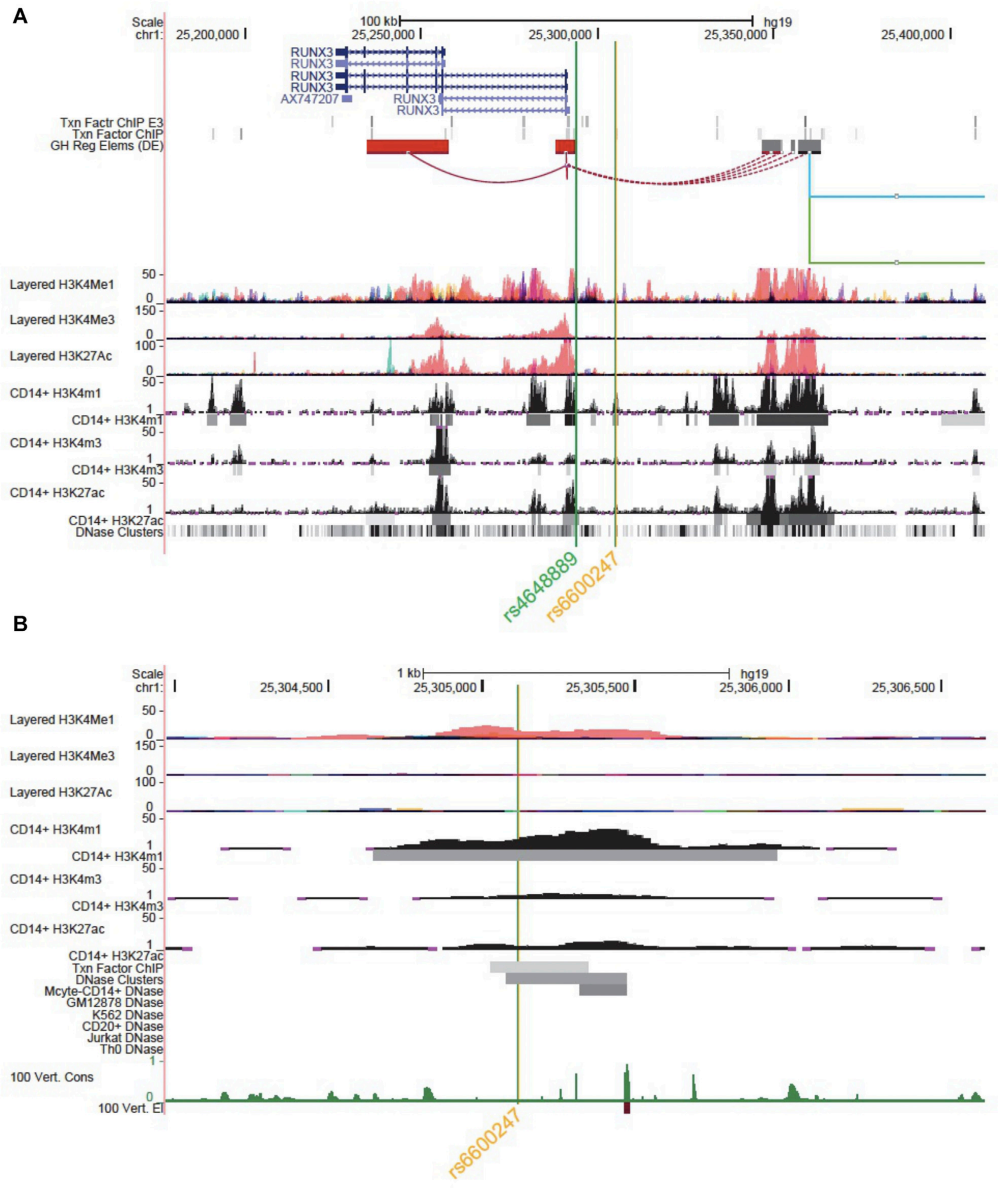
Genomic landscape interrogation suggests a regulatory role for *rs6600247*. UCSC Genome Browser analysis of the *RUNX3* locus at chr1:25,177,797–25,409,807. The green and orange vertical lines show the location of SNP *rs4648889* and *rs6600247*. *RUNX3* gene location (blue) is from the UCSC genes database. Two tracks show transcription factor binding by ChIP (Txn Facto ChIP E3 and Txn Factor ChIP). GeneHancer track shows human regulatory elements and their inferred target genes. ENCODE layered H3K27ac, H3K4Me1 and H3K4me3 tracks (from 7 cell lines) show enrichment of these marks, indicating promoter and enhancer regions. CD14^+^ H3K4me1, CD14^+^ H3K4me3 and CD14^+^ H3K27ac show enrichment and peaks called specifically in CD14^+^ monocytes (ENCODE). Dnase clusters show DNase-I hypersensitivity clusters (ENCODE); **(B)** Zoomed Genome Browser view (chr1:25,303,971–25,306,641) upstream of the promoter of *RUNX3* showing a peak for H3K4Me1 enrichment overlapping *rs6600247* (vertical line; Layered tracks and ENCODE histone tracks as in **(A)**. Additional datasets displayed are ENCODE transcription factor Chip-seq peaks and DNaseI HS peaks that directly overlap *rs6600247*, ENCODE dnase I HS for CD14^+^ monocytes and vertebrate conservation.

Figure 1A shows the genomic landscape at the *RUNX3* locus, with the lead AS-associated SNP *rs6600247* lying ∼13kb upstream of the distal promoter while the regulatory SNP *rs4648889* is physically closer to the promoter. SNP *rs6600247* is situated within a region of open chromatin, defined by a peak for dnase I hypersensitvity (DHS - indicative of regions of open chromatin) (Figure 1B), and a peak of H3K4Me1 histone modification. This sequence also binds the transcription factor c-MYC (ENCODE Factorbook (http://www.factorbook.org/human/chipseq/tf/) (Figure 1B). Taken together, these data suggest an enhancer-type element surrounding *rs6600247*, so we sought to determine a regulatory role of this SNP. The DHS peak overlapping *rs6600247* is seen specifically in CD14^+^ monocytes (Figure 1B). For this reason, we conducted our functional experiments in U937 cells, a pro-monocytic, human myeloid leukaemia cell line, exhibiting monocyte-like features.

### *rs6600247* AS-Risk C Allele Alters c-MYC Binding to Deoxyribonucleic Acid

**FIGURE 2:**
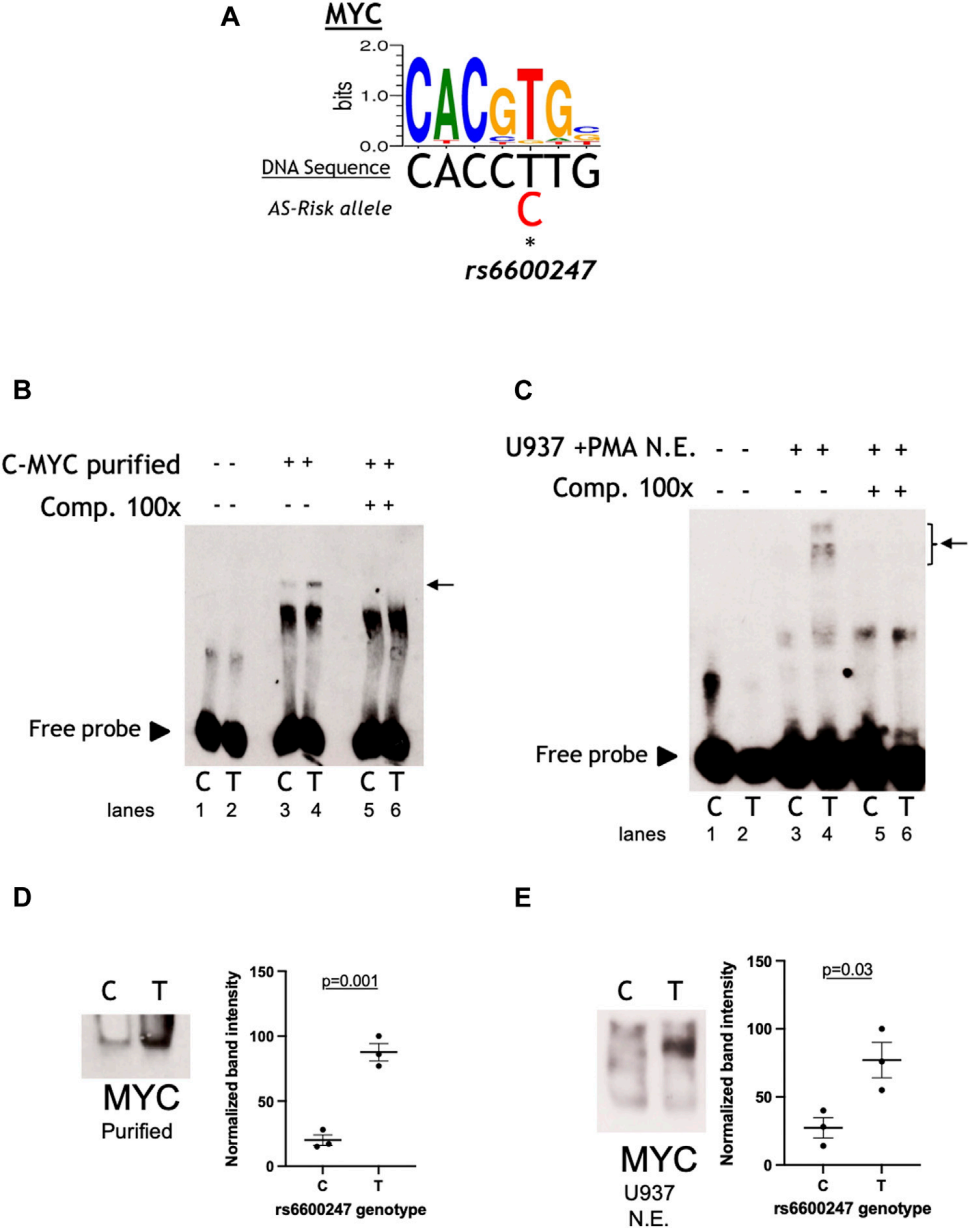
*rs6600247* risk allele affects C-Myc binding. **(A)** C-Myc binding motif analyzed using the MEME program and the location of *rs6600247* risk allele; **(B)** EMSA using c-MYC purified protein with or without specific competitor (Comp 100x), *n* = 4, allele (C or T) of *rs6600247* included in the 50bp biotinylated double-stranded DNA probe is given below the image; horizontal arrow indicates specific protein-DNA complex formation; **(C)** EMSA using nuclear extract (N.E.) from U937 cells stimulated with phorbol 12-myristate 13-acetate (PMA) with or without specific competitor (Comp 100x), *n* = 4; *rs6600247* allele and complex formation indicated as in **(B,D)** WEMSA using C-Myc purified protein and blotted with an antibody against C-Myc; **(E)** WEMSA using U937 nuclear extract and blotted with an antibody against C-Myc. The blot is representative of *n* = 3 experiments. Binding in **(D,E)** was quantified using ImageJ software and is representative of three different experiments, demonstrating that the risk allele for rs6600247 shows fewer binding properties for C-Myc.

We analysed the DNA sequence at *rs6600247* and found that the SNP lies within a c-MYC consensus binding motif (Figure 2A). We hypothesised that binding of c-MYC protein to a DNA sequence containing the risk allele C would be reduced. The results of EMSA assessing the relative c-MYC protein binding to the C or T alleles are shown in Figure 2. We first incubated probes with recombinant c-MYC purified protein, and observed a specific DNA/protein complex with both alleles but markedly less to the AS-risk allele C than the protective T allele. (Figure 2B; lane 3-4, *n* = 3). We then incubated the same probes with NE from U937 (monocyte-like cells) and observed a major protein/DNA complex binding to the protective T allele, but none with the C allele (Figure 2C, lanes 3-4, *n* = 3). In both cases, successful competition with a 100-fold excess of unlabelled probe confirmed the specificity of the complex (Figure 2B, lane 5-6 and Figure 2C lane 5–6). We next used WEMSA to quantitate the relative binding of c-MYC to each allele of *rs6600247.* Markedly less c-MYC enrichment was seen with the C risk vs T allele using either c-MYC purified protein or U937 NE (Figures 2D,E, relative band intensities *p* = 0.01 and *p* = 0.05, respectively, two-sample *t* test). We also repeated these experiments using CD8^+^ T-cells and Jurkat NE, showing no differential binding between the two alleles (Supplementary Figure S1).

### The *RUNX3* Promoter Interacts With the *rs4648889* Region Rather Than *rs6600247*

**FIGURE 3:**
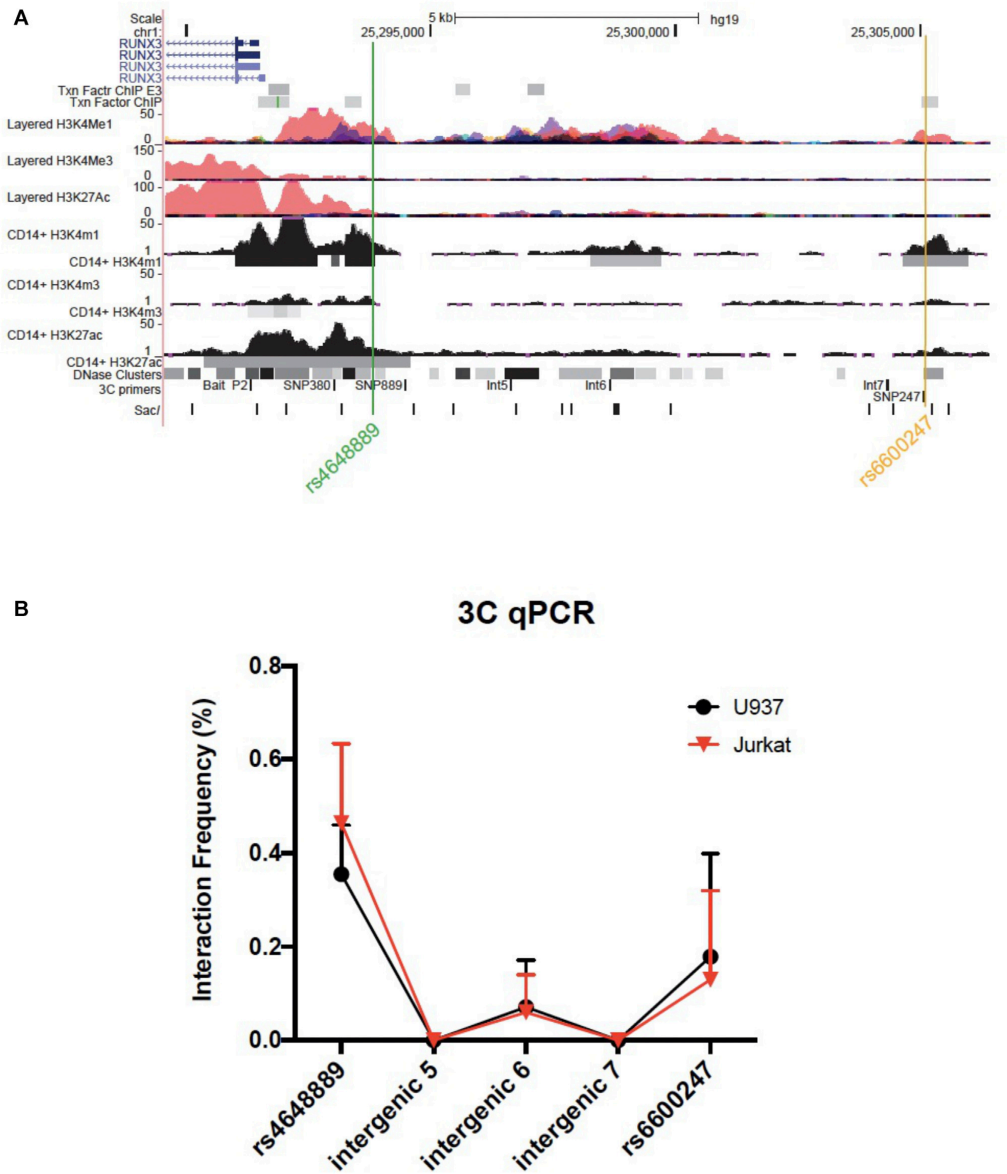
Chromosome looping investigation demonstrates interaction between the *RUNX3* promoter and region encompassing *rs4648889* rather than *rs6600247*. **(A)** Location of the *RUNX3* genomic region chr1:25,289,567–25,306,400. Tracks shown as in Figure 1, with the addition of 3C-qPCR primers and SacI enzyme cutting sites. Bait fragment is located at *RUNX3* distal promoter (P2); AS-associated SNPs primers used in these experiments are named as follows: SNP889 (for *rs4648889*), Int5, Int6, Int7 (for intergenic regions 5, 6 and 7) and SNP247 (for *rs6600247*); **(B)** Results of the 3C-qPCR analysis showing Increased relative interaction frequency between *RUNX3* P2 and the region encompassing *rs4648889,* with a modest interaction with the *rs6600247* region. Theses interactions were seen in both U937 (red) and Jurkat (black) cell lines.

We used 3C to test plausible chromosome looping interactions between the AS-associated SNP *rs6600247* and the *RUNX3* distal promoter. Figure 3A shows the RUNX3 genomic region interrogated, the location of the primers and Sac1 restriction sites. Baits were designed to capture Sac1 fragments containing *rs6600247,* three intergenic fragments with H3K4me1 enrichment, and additionally with a previously-studied AS-associated SNP *rs4648889*. There was very low interaction frequency between *rs6600247* and the distal *RUNX3* promoter, either in U937 or Jurkat cells. A stronger interaction frequency was observed between the distal promoter and the region encompassing the AS-associated SNP *rs4648889* (Figure 3B) confirming its functional role.

### *rs6600247* Genotype has No Effect on *RUNX3* Expression

**FIGURE 4:**
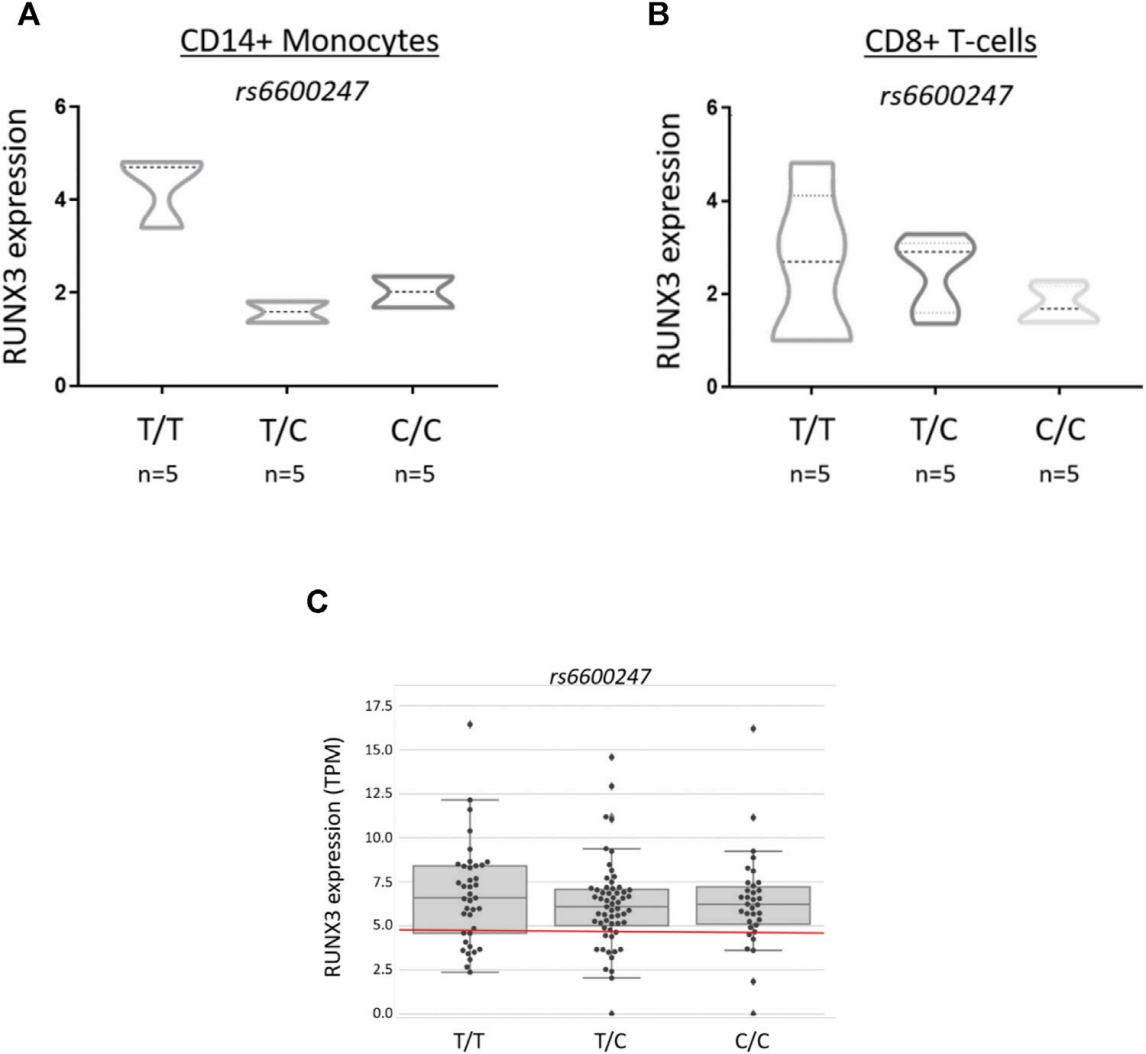
*rs6600247* genotype shows no regulatory effect on RUNX3 expression *RUNX3* expression levels measured by qRT-PCR in freshly isolated **(A)** CD14^+^ monocytes and **(B)** CD8^+^ T-cells from 15 AS patients stratified according to *rs6600247* genotype. Statistical analysis performed with Welch’s two-sample *t* test; **(C)** Expression of *RUNX3* in an historical RNA-seq dataset (Li et al., 2017) obtained from PBMCs, stratified on *rs6600247*.

Primary CD14^+^ monocytes and CD8^+^ T-cells from AS patients were used to evaluate *RUNX3* mRNA expression stratified on *rs6600247* genotype (*n* = 5 each genotype) (Figures 4A,B). There was a non-significant trend for lower expression in CD14^+^ monocytes with the AS-risk CC genotype compared to protective TT and heterozygous TC genotypes (TT vs CC: 4.6 ± 1.8 vs 2.0 ± 0.4; TT vs CT: 4.6 ± 1.8 vs 1.8 ± 0.2; CC vs CT: 2.0 ± 0.4 vs 1.8 ± 0.2, results are expressed as mean ± standard error mean). We also analysed historical RNA-seq data (Li et al., 2017) obtained from AS case PBMCs measuring *RUNX3* mRNA expression, stratified on *rs6600247*: there was no apparent influence from this SNP on *RUNX3* expression (Figure 4C).

## Discussion

In this study, we have demonstrated that the lead AS-associated SNP in the *RUNX3* region, *rs6600247,* affects the binding of c-MYC to the region of DNA 13 kb upstream of the *RUNX3* promoter, which lies in a region of open chromatin in CD14^+^ monocytes. Further, this region showed enrichment for H3K4Me1 modification in the absence of H3K4me3 or H3K27ac enrichment, suggesting a weak or poised enhancer (Gasperini et al., 2020). Although GWAS have identified hundreds of genetic variants associated with AS (Cortes et al., 2013), only a very small portion of these have been investigated to define causal variants. Cell type and stimulation conditions must be taken carefully in consideration in identifying causal SNPs, as both impact on chromatin interaction and gene regulation (Shi et al., 2021).

Recent findings have shown that RUNX3 is highly expressed in monocytes where it has a role in transcriptional repression, metabolic regulation, and in tuning the function of CD14^+^ monocytes. (Puig-Kröger et al., 2010; Estecha et al., 2012). Expression of RUNX3 has been found also in CD11c + mature intestinal macrophages, suggesting a role for this TF in macrophage maturation (Corbin et al., 2020). Further, both RUNX3 and another TF, ID2 (Inhibitor of DNA binding 2), are required for the differentiation of epidermal Langerhans cells from monocytes (Fainaru et al., 2004).

The interaction between RUNX3 and c-MYC has previously been investigated in T-cell lymphoma (Selvarajan et al., 2017). Double immunofluorescence revealed co-localization of both proteins in the tumour nuclei. In addition, several binding-sites for c-MYC were identified in the RUNX3 enhancer region. Additional evidence for this interaction stems from colorectal cancer studies where upregulation of RUNX3 by Bone Morphogenetic Protein (BMP) reduces c-MYC expression, thereby exerting c-MYC tumour-suppressor activity (Lee et al., 2010). Recently, it has been demonstrated that two super-enhancers located at 59 and 70 kb upstream of the RUNX3 transcription start site are required for both RUNX3 and MYC expression and function (Hosoi et al., 2021). Other studies also indicate a key role of c-MYC in monocyte/macrophage activation, as it is involved in the regulation of different alternative activation genes (Pello et al., 2012).

Our EMSA/WEMSA experiments confirmed c-MYC binding at the *rs6600247* locus, with the AS-risk allele disrupting the binding motif and consequently reducing formation of the c-MYC-DNA complex. Altogether, these observations are consistent with the hypothesis that c-MYC can bind the *RUNX3* promoter and/or regulatory elements upstream of the promoter thereby potentially playing a role in the regulation of *RUNX3*. The processes involved in transcriptional regulation are complex and this finding does not exclude the possibility of other TFs being involved.

The genome is organized in a very dynamic way and TFs mediate chromosome loops to bring enhancers and promoters together (de Wit and de Laat, 2012; Palstra and Grosveld, 2012). 3C and related techniques are the classic approach to demonstrating interactions between target genes and enhancers or enhancer-like regions. Here we demonstrate the presence of chromatin loop between a SNP overlapping a regulatory region and the distal promoter of *RUNX3* using 3C followed by qPCR. This method has been used extensively to demonstrate interactions between various regulatory regions in different cell types and it allows one to quantitate the interaction frequency (Sati and Cavalli, 2017; McCord et al., 2020).

We accept that recent findings highlight the fact that contact frequencies from 3C assays sometimes do not correspond to 3D proximity (Williamson et al., 2014), but taken together with the functional data presented here and other recently published findings (Vecellio et al., 2016; Vecellio et al., 2018; Vecellio et al., 2021) we are confident in our results. However, we are aware that other higher throughput techniques have been developed (eg. 4C, 5C, Hi-C) that might give a more general overview of the regulation of the *RUNX3* locus and the genetic interactions of the SNPs in this region. These will be incorporated into our ongoing genome-wide studies of chromatin interactions and the regulatory effects of AS-associated genetic variants.

Here, we have demonstrated physical interactions between the distal promoter of *RUNX3* and *rs4648889* SNP, which we have previously functionally characterized (Vecellio et al., 2016; Vecellio et al., 2021). Conversely, there was a very low interaction frequency with *rs6600247* that suggests no functional role for this SNP in chromosome looping in the particular context of CD8^+^ T-cells or monocytes. As previously shown, in a ∼15 kb linkage disequilibrium (LD) block upstream the promoter of *RUNX3*, there are 22 *RUNX3* SNPs that are strongly associated with AS (*p* ≤ 10^–14^) (Vecellio et al., 2016). The SNP analysed in this work, *rs6600247* (*p* = 1.3 × 10^–14^), is in complete LD with *rs4648889* (∼2 kb upstream of the *RUNX3* promoter). Conditional analysis established the primacy of the *rs4648889* association with AS at *RUNX3* (Vecellio et al., 2016), while not excluding additional functional roles for other SNPs in LD with it. The functional experiments described here and in previous publications (Vecellio et al., 2016; Vecellio et al., 2018; Vecellio et al., 2021) represent an approach to identifying more precisely which SNPs in this LD block actually have a functional impact on the *RUNX3* regulatory element and its role in the pathogenesis of AS.

Clearly the presence of an enhancer-promoter loop alone does not ensure activation of a target gene but it provides a platform where transcription factors can bind and regulate gene/s (Espinola et al., 2021; Ing-Simmons et al., 2021). Here, we have confirmed that the genomic regulatory element upstream of the *RUNX3* promoter has potentially important cell-type-specific functional effects. We show that the *rs6600247* AS-risk allele affects c-MYC binding in monocytes, suggesting that c-MYC/RUNX3 modulated pathways could have a role in the pathophysiology of AS. Nevertheless, the region encompassing *rs6600247* has no significant physical interaction with the distal *RUNX3* promoter, thereby confirming that *rs4648889* appears to be the cardinal genetic variant associated with AS at the *RUNX3* locus.

Further studies are required to identify additional higher order chromatin interactions at this locus. For example, HiChIP has been used to delineate promoter-enhancer interactions in keratinocytes and CD8^+^ T-cell lines exploring psoriasis and psoriatic arthritis disease-associated SNPs and similar methods could be explored in AS (Shi et al., 2020a; Shi et al., 2020b). It is also critical that cell-type and -context specificity are crucial for TF binding and activity, and can also influence chromatin looping data (Nancy et al., 2021). While we have presented here and elsewhere evidence for the involvement of CD8^+^ T-cells and monocytes in the pathogenesis of AS other cell types must also be considered. These include numerous types found in bone and cartilage, various other components of the immune system and also cells in the gut where chronic low-grade inflammation is a feature of AS in around two-thirds of cases (Ciccia et al., 2016; Shao et al., 2021). In the future targeted *RUNX3* enhancer element genomic editing strategies could be used to elucidate their effects on RUNX3 (and other gene) expression and downstream cellular signaling.

In conclusion, this work provides new insights into the complex transcriptional regulation of *RUNX3* and the role that AS-associated SNPs may play in this process. We highlight the importance of functional studies in determining which disease associated SNPs are primarily involved in the pathogenesis of such diseases and the importance of interrogating their role in the appropriate cellular context.

## Data Availability

The raw data supporting the conclusions of this article will be made available by the authors, without undue reservation.
