# Supplementary figures and images for: Disruption of c-MYC Binding and Chromosomal Looping Involving Genetic Variants Associated With Ankylosing Spondylitis Upstream of the RUNX3 Promoter
Source: Front Genet. 2022 Jan 7;12:741867. doi: 10.3389/fgene.2021.741867 (PMC8782160; doi:10.3389/fgene.2021.741867)

# Suppl Fig.1

**a**

|                   |   |   |   |   |   |   |
|-------------------|---|---|---|---|---|---|
| CD8+ T-cells N.E. | - | - | + | + | + | + |
| Comp. 100x        | - | - | - | - | + | + |

Free probe ►

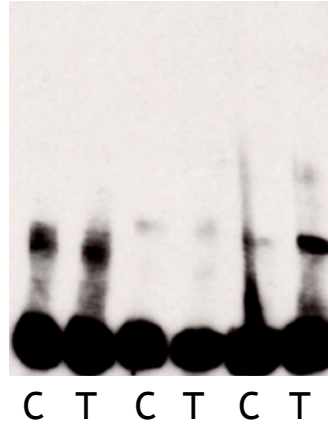

**b**

|             |   |   |   |   |   |   |
|-------------|---|---|---|---|---|---|
| Jurkat N.E. | - | - | + | + | + | + |
| Comp. 100x  | - | - | - | - | + | + |

Free probe ►

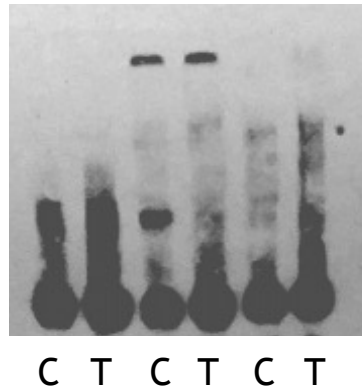

**c**

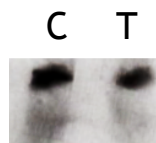

MYC  
CD8  
N.E.

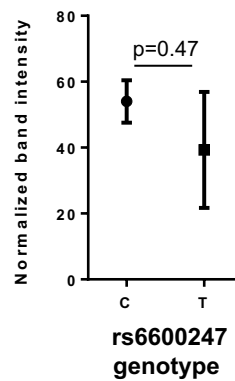

Supplement: Supplementary file 2 [file Image1.pdf]
